# Immunochemical Approach for Monitoring of Structural Transition of ApoA-I upon HDL Formation Using Novel Monoclonal Antibodies

**DOI:** 10.1038/s41598-017-03208-8

**Published:** 2017-06-07

**Authors:** Hitoshi Kimura, Shiho Mikawa, Chiharu Mizuguchi, Yuki Horie, Izumi Morita, Hiroyuki Oyama, Takashi Ohgita, Kazuchika Nishitsuji, Atsuko Takeuchi, Sissel Lund-Katz, Kenichi Akaji, Norihiro Kobayashi, Hiroyuki Saito

**Affiliations:** 1 Department of Biophysical Chemistry, Kyoto Pharmaceutical University, 5 Nakauchi-cho, Misasagi, Yamashina-ku, Kyoto 607-8414 Japan; 2 Graduate School of Pharmaceutical Sciences, Tokushima University, 1-78-1 Shomachi, Tokushima, 770-8505 Japan; 3 Department of Bioanalytical Chemistry, Kobe Pharmaceutical University, 4-19-1 Motoyama-Kitamachi, Higashinada-ku, Kobe 658-8558 Japan; 4 Department of Molecular Pathology, Institute of Biomedical Sciences, Tokushima University Graduate School, 3-18-15 Kuramoto-cho, Tokushima, 770-8503 Japan; 5 Analytical Laboratory, Kobe Pharmaceutical University, 4-19-1 Motoyama-Kitamachi, Higashinada-ku, Kobe 658-8558 Japan; 6 Lipid Research Group, Gastroenterology, Hepatology and Nutrition Division, The Children’s Hospital of Philadelphia, Perelman School of Medicine at the University of Pennsylvania, Philadelphia, Pennsylvania 19104-4318 USA; 7 Department of Medicinal Chemistry, Kyoto Pharmaceutical University, 5 Nakauchi-cho, Misasagi, Yamashina-ku, Kyoto 607-8414 Japan

## Abstract

Apolipoprotein A-I (apoA-I) undergoes a large conformational reorganization during remodeling of high-density lipoprotein (HDL) particles. To detect structural transition of apoA-I upon HDL formation, we developed novel monoclonal antibodies (mAbs). Splenocytes from BALB/c mice immunized with a recombinant human apoA-I, with or without conjugation with keyhole limpet hemocyanin, were fused with P3/NS1/1-Ag4-1 myeloma cells. After the HAT-selection and cloning, we established nine hybridoma clones secreting anti-apoA-I mAbs in which four mAbs recognize epitopes on the N-terminal half of apoA-I while the other five mAbs recognize the central region. ELISA and bio-layer interferometry measurements demonstrated that mAbs whose epitopes are within residues 1–43 or 44–65 obviously discriminate discoidal and spherical reconstituted HDL particles despite their great reactivities to lipid-free apoA-I and plasma HDL, suggesting the possibility of these mAbs to detect structural transition of apoA-I on HDL. Importantly, a helix-disrupting mutation of W50R into residues 44–65 restored the immunoreactivity of mAbs whose epitope being within residues 44–65 against reconstituted HDL particles, indicating that these mAbs specifically recognize the epitope region in a random coil state. These results encourage us to develop mAbs targeting epitopes in the N-terminal residues of apoA-I as useful probes for monitoring formation and remodeling of HDL particles.

## Introduction

Plasma levels of high-density lipoprotein (HDL) cholesterol are well known to be associated with a reduced risk of cardiovascular disease^1, 2^. The anti-atherogenic properties of HDL arise, in part, from its participation in the reverse cholesterol transport pathway in which the principal protein, apolipoprotein A-I (apoA-I), plays a central role^3, 4^. It is becoming apparent that the anti-atherogenic effects of HDL are not only dependent on its concentration in plasma but also on its biological functionality^5–7^ such as cholesterol efflux capacity from cells^8, 9^ and apoA-I exchangeability in which apoA-I can dissociate from the HDL surface and exchange between HDL particles^10, 11^.

HDL particles are quite heterogeneous in their size, shape, and lipid and protein compositions^12, 13^. The heterogeneity of HDL particles primarily comes from the highly dynamic structure of apoA-I^14^, allowing it to adapt multiple lipid-bound conformations on HDL particles of different sizes and lipid and/or protein compositions^3, 15–17^. Upon HDL formation, apoA-I molecule undergoes a large conformational reorganization in which the opening of the N-terminal helix bundle occurs, converting hydrophobic helix-helix interactions to helix-lipid interactions^3, 15, 18, 19^. The apoA-I molecules in an anti-parallel, double-belt conformation stabilize nascent discoidal HDL particles of different sizes with certain segments forming flexible loops that detach reversibly from the surface^20–22^. In mature spherical HDL particles, the apoA-I molecules in the double-belt conformation bend and form a stabilizing trefoil scaffold structure^16, 23^. In addition, such conformational plasticity and flexibility of apoA-I are also thought to be associated with its strong amyloidogenic property^24, 25^.

To discriminate the different apoA-I conformations on HDL particles, native monoclonal antibodies (mAbs) generated by hybridoma techniques targeting epitopes distributed along the apoA-I sequence^26–32^ as well as recombinant antibody fragments isolated from phage-displayed libraries^33^ have been used. A mAb that specifically recognizes an epitope of apoA-I exposed only in preβ1-HDL (or lipid-poor apoA-I) has been commercially available to measure preβ1-HDL concentration in plasma^34, 35^. In addition, mAbs that specifically recognize oxidized^36, 37^ or nitrated^38^ apoA-I in both lipid-free and HDL-bound forms have been developed to assess the distribution and function of modified, dysfunctional apoA-I in plasma as well as in artery wall.

We previously developed a novel method for assessing HDL production from cells based on the lipidation-induced hydrophobicity change in apoA-I during HDL formation^39^. In this assay, we took advantage of a significant increase in fluorescence intensity when a fluorescence-labeled helix in apoA-I transfers from an aqueous environment to the hydrophobic lipid surface^11, 15^. In the present study, we focused on the fact that apoA-I molecule undergoes a large conformational transition in which the random coil regions both in the N-terminal and C-terminal domains form α-helical structure upon lipid binding^19, 21, 40, 41^. To detect the secondary structural transition, we generated a panel of novel mAbs against human apoA-I, and found that mAbs that target epitopes in the N-terminal regions of apoA-I can recognize structural transition of apoA-I upon formation of HDL particles. The present results suggest that these mAbs may provide useful tools for monitoring formation and remodeling of HDL particles.

## Results and Discussion

### Generation of anti-apoA-I mAbs

Several reports have been published from the 1980s^42–45^ for generation of anti-apoA-I mAbs based on the hybridoma method^46^. In most of these studies, mice were immunized with native apoA-I or HDL isolated from human plasma without conjugation with carrier macromolecules. In our present study, BALB/c mice, the most common splenocyte donor used for the hybridoma production, were immunized with recombinant apoA-I expressed in *Esherichia coli* (*E*. *coli*) cells. Augmentation of its immunogenicity was also attempted by conjugation with keyhole limpet hemocyanin (KLH), which is a well-known carrier protein.

**Figure 1:**
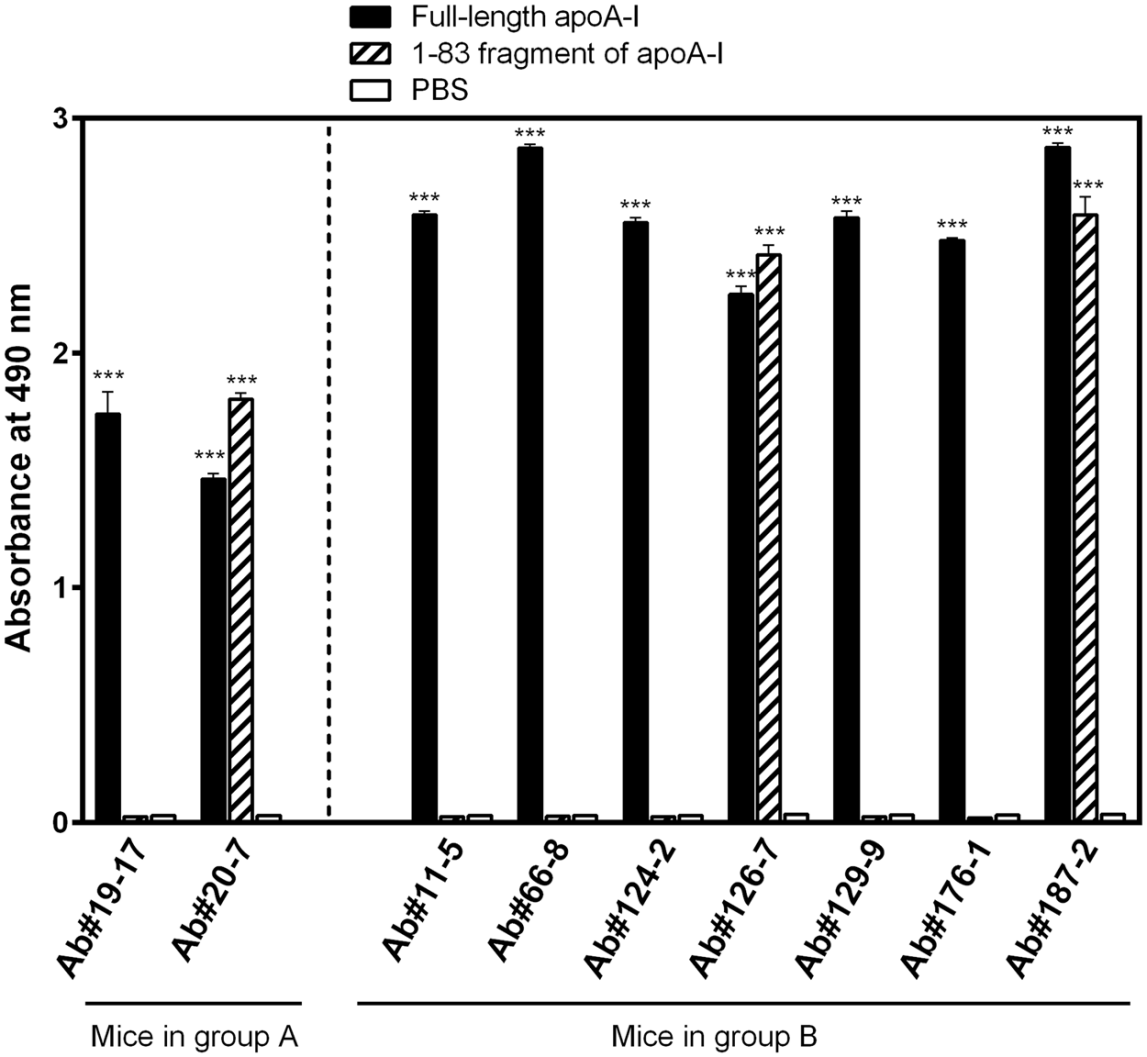
Reactivity of the present monoclonal antibodies to full-length and the N-terminal 1–83 fragment of apoA-I. Biotinylated full-length apoA-I or its N-terminal 1–83 fragment (0.3 μM) in G-PBS, or PBS (for examining the background value) was added to 96-well microplates coated with streptavidin, and then incubated for 30 min at 37 °C. After washing the wells with T-PBS, hybridoma supernatants, 500-fold diluted with G-PBS, were added to the wells (100 μl/well) and incubated at 37 °C for 30 min. After washing, POD-conjugated anti-mouse IgG antibody diluted at 1:5000 in G-PBS (100 μl/well) was added and incubated for 30 min at 37 °C. The wells were washed and bound POD activity was determined colorimetrically (see Materials and Methods). ***P < 0.001 versus PBS.

Ten mice were divided into two groups (A and B), and repeatedly immunized with apoA-I (group A) or apoA-I-KLH conjugate (group B) (see Supplementary Information). Unexpectedly, no significant difference was observed in serum titer for anti-apoA-I antibodies between the group A and B (Fig. S1, *P* = 0.93). The first fusion experiment was performed using splenocytes from the mouse (#5) (see Fig. S1) in group A. Initial screening by the ELISA using microplates coated with the apoA-I-BSA conjugate (see Supplementary Information) showed that more than 35 micro-cultures (from a total of 576 cultures) were antibody-positive. However, only two kinds of hybridoma clones, each secreting the antibodies Ab#19–17 or Ab#20–7 (Fig. 1), were finally found to be useful for this study.

The second fusion used the mice in group B (#2 and #4) as spleen donor. Final immunization was performed with apoA-I without KLH conjugation in order to preferentially stimulate B-cell clones that recognize epitopes on intact apoA-I molecules. Screening of the initial hybridoma cultures (total 968) were carried out by the ELISA using biotin-labeled apoA-I to avoid the possibility that BSA might impair the structures of epitopes inherent to native apoA-I. This fusion provided 192 antibody-positive micro-cultures, and finally seven kinds of hybridoma clones secreting antibodies that show strongly positive signals in ELISA (Ab#11-5, Ab#66-8, Ab#124-2, Ab#126-7, Ab#129-9, Ab#176-1, and Ab#187-2). We note that, among these nine mAbs, Ab#19-17 and Ab#20-7 were already applied for our recently published studies^39, 47, 48^.

### Fundamental characterization of anti-apoA-I mAbs

Isotyping revealed that all the nine mAbs are composed of *κ* light chains in the combination with *γ*1 (Ab#20-7, Ab#66-8, Ab#126-7, Ab#129-9, Ab#176-1, and Ab#187-2) or *γ*2a (Ab#11-5, Ab#19-17, and Ab#124-2) heavy chains. Among these mAbs, Ab#20-7, Ab#126-7, and Ab#187-2 reacted almost equally to the recombinant full-length and the N-terminal 1–83 fragment of apoA-I, while the other six mAbs reacted only to full-length apoA-I (Fig. 1). We previously demonstrated that the N-terminal 1–83 fragment of apoA-I has a strong propensity to form amyloid fibrils^49, 50^, which is the hallmark of apoA-I amyloidogenesis: for this reason we tested here the reactivities of the mAbs to this fragment. The results suggested that these three antibodies recognize epitopes within the N-terminal 1–83 residues whereas the other six mAbs should recognize epitopes out of 1–83 residues in apoA-I. We note that all nine mAbs established here reacted to native apoA-I purified from human plasma as well as the recombinant apoA-I tested here (Fig. S2).

### Epitope determination of anti-apoA-I mAbs

**Figure 2:**
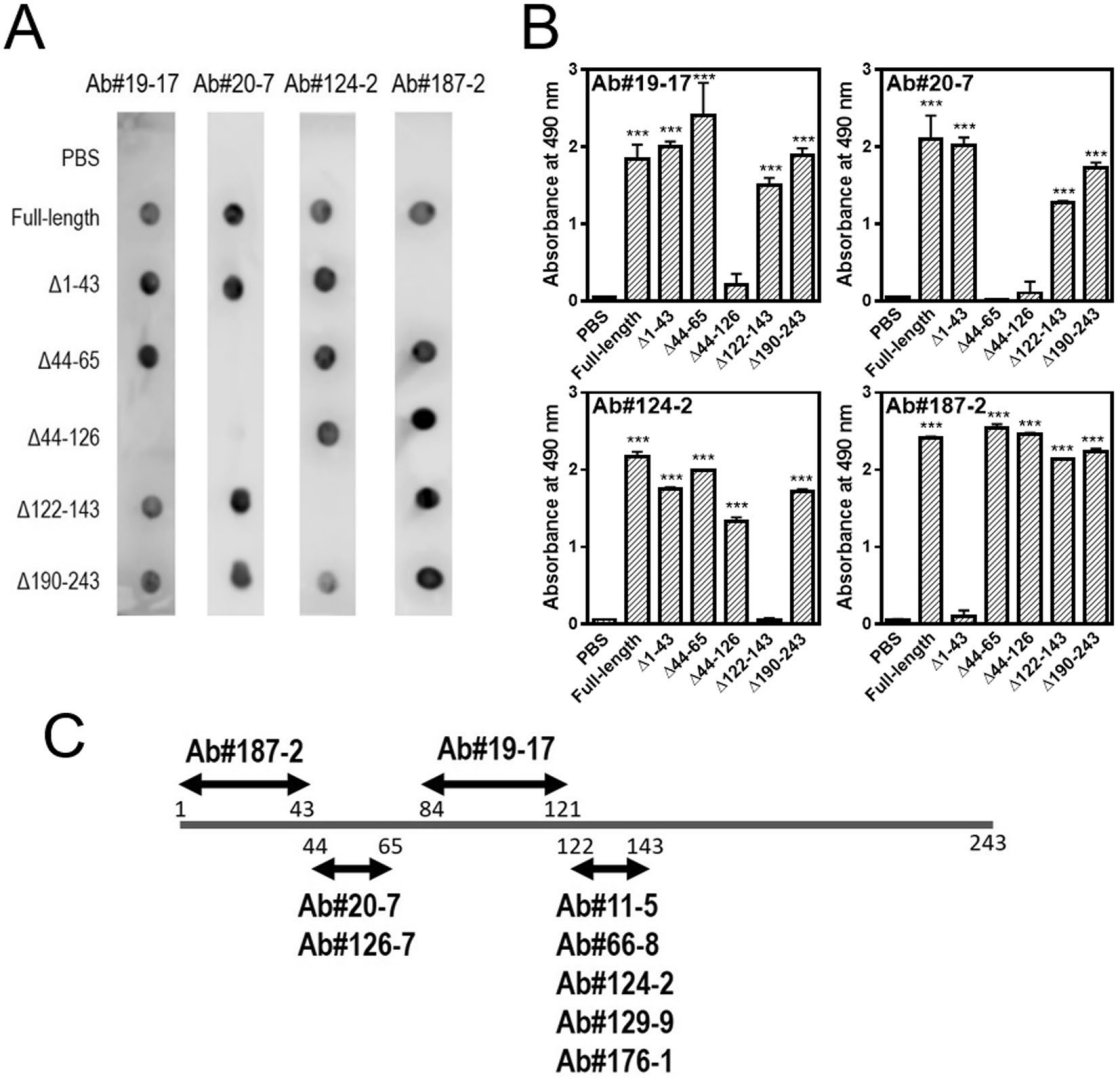
Epitope determination of anti-apoA-I mAbs. (**A**) ApoA-I deletion variants were blotted on nitrocellulose membranes with Ab#19-17, Ab#20-7, Ab#124-2, and Ab#187-2. (**B**) Ab#19-17, Ab#20-7, Ab#124-2, or Ab#187-2 were added to 96-well microplates coated with biotinylated apoA-I deletion variants, and detected with anti-mouse IgG antibody. (**C**) Schematic diagram of the epitope-containing regions in apoA-I recognized by mAbs. ***P < 0.001 versus PBS.

To identify epitopes of anti-apoA-I mAbs we generated, a series of deletion mutants of apoA-I lacking N-terminal (∆1–43, ∆44–65, and ∆44–126), central (∆122–143), or C-terminal (∆190–243) regions along the molecule were used^18^. Figure 2A shows the typical results of dot blotting of mAbs against apoA-I deletion mutants trapped on the membrane. Ab#19-17, Ab#20-7, Ab#124-2, and Ab#187-2 did not recognize ∆44–126, ∆44–65 and ∆44–126, ∆122–143, and ∆1–43 apoA-I, respectively, indicating that epitopes of Ab#19-17, Ab#20-7, Ab#124-2, and Ab#187-2 are within residues 66–121, 44–65, 122–143, and 1–43, respectively. Since Ab#19-17 did not recognize the 1–83 fragment of apoA-I (Fig. 1), the epitope of Ab#19-17 is considered to be within residues 84–121.

We also examined the reactivity of mAbs against apoA-I deletion mutants by a capture ELISA in which biotinylated apoA-I deletion mutants were coated on ELISA plates. Consistent with the results of dot blotting, each mAb exhibited negligible reactivity only against apoA-I deletion mutants lacking the expected epitope regions (Fig. 2B). We also found that the epitope for Ab#126-7 is within residues 44–65 (Fig. S5A) whereas those for Ab#11-5, #66-8, Ab#129-9, and Ab#176-1 are within residues 122–143 (data not shown). Based on these results, the epitope-containing regions in the apoA-I molecule recognized by each mAbs are schematically summarized in Fig. 2C.

### Reactivity of mAbs against apoA-I in lipid-free or HDL-bound forms

We next investigated effects of lipidation of apoA-I on the reactivity of mAbs. To this end, biotinylated Cys-apoA-I in lipid-free form or reconstituted 1-palmitoyl-2-oleoylphosphatidylcholine (POPC) discoidal complexes was immobilized on the streptavidin-coated plates. To avoid the possible impairing effects of biotinylation of lysine residues in apoA-I on its lipid binding ability^51^, we used a N-terminally biotinylated Cys-apoA-I because the N-terminal 1–6 residues are not involved in formation of α-helical structure of apoA-I on discoidal HDL particles^21^.

**Figure 3:**
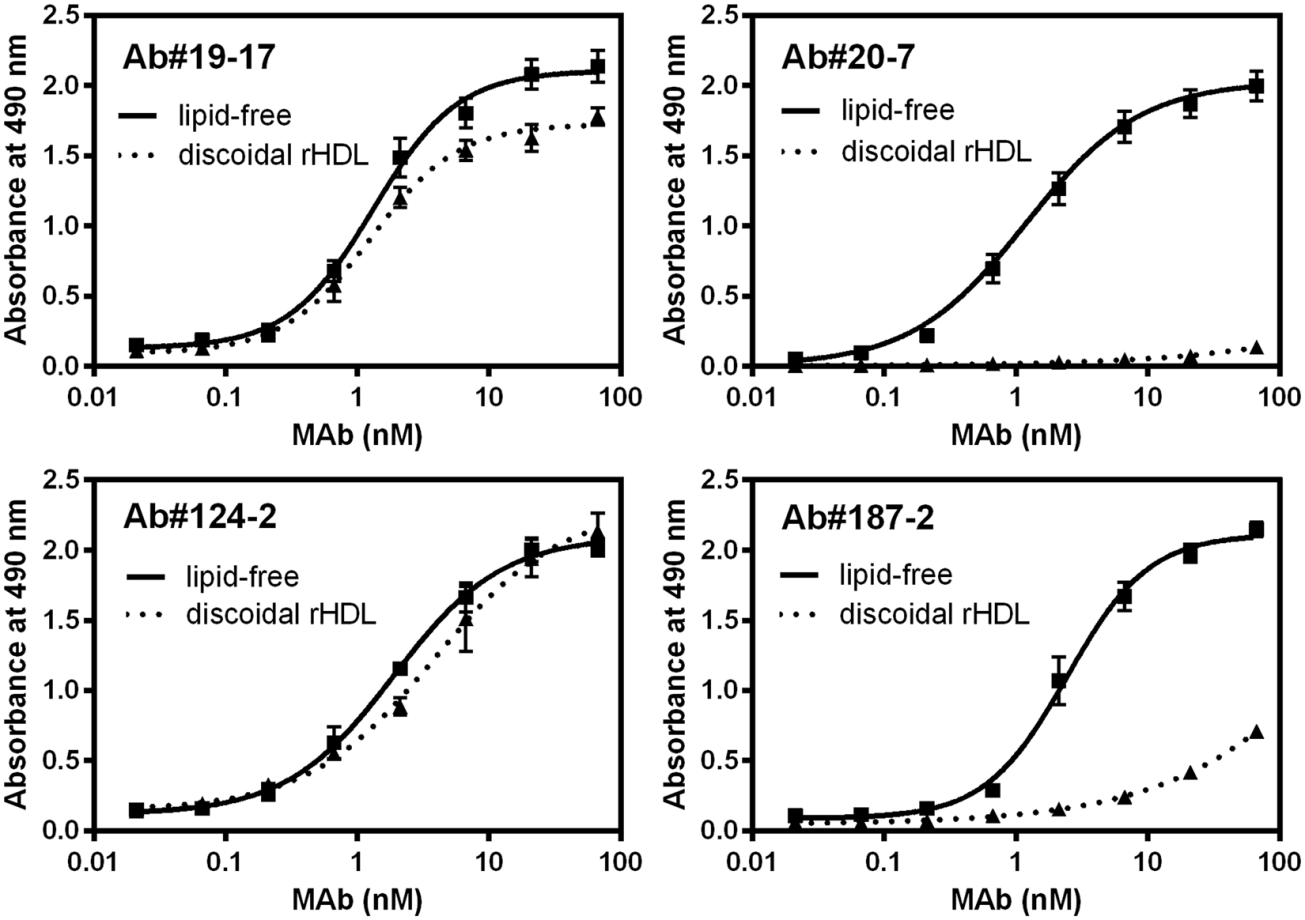
Reactivity of mAbs against apoA-I in the lipid-free state or on reconstituted discoidal HDL by ELISA. Biotinylated Cys-apoA-I in the lipid-free state or on discoidal HDL were added to 96-well microplates coated with streptavidin. After washing, Ab#19-17, Ab#20-7, Ab#124-2, or Ab#187-2 were added to the plates with increasing concentrations, and then detected with POD-conjugated anti-mouse IgG antibody.

As shown in Fig. 3, all mAbs reacted with lipid-free apoA-I in a concentration-dependent manner, in which dissociation constant *K*_D_ values determined from the fitting to sigmoidal does-response curves were in the range of 1.2–2.5 nM (Table 1). In contrast, although Ab#19-17 and Ab#124-2 reacted with apoA-I on discoidal complexes similarly to that in the lipid-free form, Ab#20-7 and Ab#187-2 exhibited deficient reactivity against disc-bound apoA-I. These results suggest that Abs#20-7 and Ab#187-2 cannot recognize epitope regions of apoA-I (within residues 1–43 and 44–65 for Ab#187-2 and Ab#20-7, respectively) in the lipidated state.

**Table 1:** *K*_D_ values (nM) for single-antibody ELISA shown in Fig. 3.

mAb	lipid-free apoA-I	discoidal rHDL
Ab#19-17	1.3 ± 0.2	1.3 ± 0.2
Ab#20-7	1.2 ± 0.2	N.D.
Ab#124-2	1.9 ± 0.3	3.8 ± 1.2
Ab#187-2	2.5 ± 0.3	N.D.

**Figure 4:**
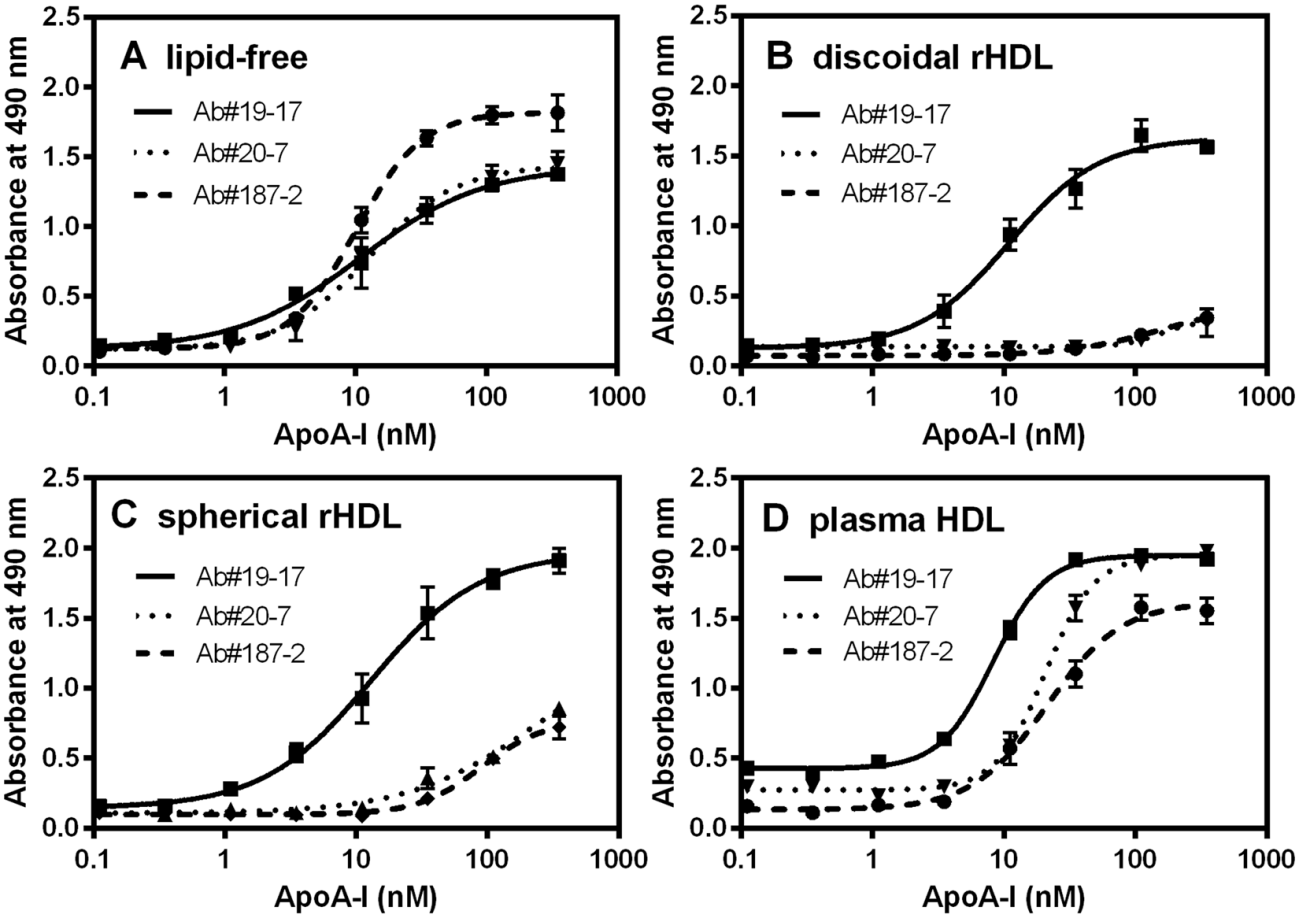
Sandwich ELISA for detection of lipid-free and HDL-bound apoA-I. ApoA-I in the lipid-free state (**A**), on reconstituted discoidal HDL (**B**) and spherical HDL (**C**), and human plasma HDL (**D**) were added to the plates coated with Ab#124-2. After washing, biotinylated Ab#19-17, Ab#20-7, or Ab#187-2 were added to the plates, and then detected with POD-conjugated streptavidin.

To confirm the specificity of Ab#20-7 and Ab#187-2 for recognition of apoA-I, a sandwich ELISA system to detect non-labeled apoA-I in the lipid-free or HDL-bound states was established. As a capture antibody, Ab#124-2 was selected because this mAb recognizes apoA-I both in the lipid-free state and on reconstituted discoidal HDL equivalently (Fig. 3). Thus, Ab#19-17, Ab#20-7, and Ab#187-2 were biotinylated and used as detection antibodies. These three mAbs reacted with lipid-free apoA-I specifically with *K*_D_ values of 10–13 nM (Fig. 4A and Table 2). In contrast, Ab#20-7 and Ab#187-2 showed obviously defective reactivity to apoA-I in reconstituted discoidal or spherical HDL particles whereas Ab#19-17 reacted similarly with apoA-I in either lipid-free or lipidated states (Fig. 4B and C). These results are consistent with the notion that Ab#20-7 and Ab#187-2 recognize each epitope region of apoA-I only in the lipid-free structure.

**Table 2:** *K*_D_ values (nM) for sandwich ELISA shown in Fig. 4.

mAb	lipid-free apoA-I	discoidal rHDL	spherical rHDL	plasma HDL
Ab#19-17	11 ± 2.9	11 ± 2.2	13 ± 3.0	8.2 ± 0.4
Ab#20-7	13 ± 2.2	N.D.	N.D.	21 ± 1.2
Ab#187-2	10 ± 1.0	N.D.	N.D.	21 ± 3.2

These three mAbs, however, recognized plasma HDL, among which Ab#20-7 and Ab#187-2 exhibited less binding affinity (*K*_D_ of 21 nM) than Ab#19-17 (*K*_D_ of 8.2 nM), as shown in Fig. 4D and Table 2. Bio-layer interferometry (BLI) analyses using biotinylated mAbs immobilized on streptavidin sensor chips, which allow us to monitor the association and dissociation kinetics for interactions of mAbs with apoA-I^52^, also demonstrated that the three mAbs exhibit binding behaviors to lipid-free apoA-I and plasma HDL with nanomolar *K*_D_ values despite the deficient binding of Ab#20-7 and Ab#187-2 to reconstituted discoidal HDL particles (Fig. S3 and Table S1). The reason why Ab#20-7 and Ab#187-2 recognize plasma HDL might be that apoA-I conformation on plasma HDL is partially unfolded, locally different from that in reconstituted HDL particles^16^. Indeed, circular dichroism (CD) spectrometry showed that the secondary structure of apoA-I on plasma HDL was significantly less helical than that in reconstituted discoidal or spherical HDL particles (Fig. S4A)^53^. It should be noted that such difference in secondary structure of apoA-I does not necessarily lead to alteration of size distribution of HDL particles^16^. Thus, it is possible that the epitope regions of Ab#20-7 (within residues 44–65) and Ab#187-2 (within residues 1–43) in apoA-I are partially unfolded in the plasma HDL used in this study.

### Effect of a helix-disrupting mutation in apoA-I on mAb recognition activity

**Figure 5:**
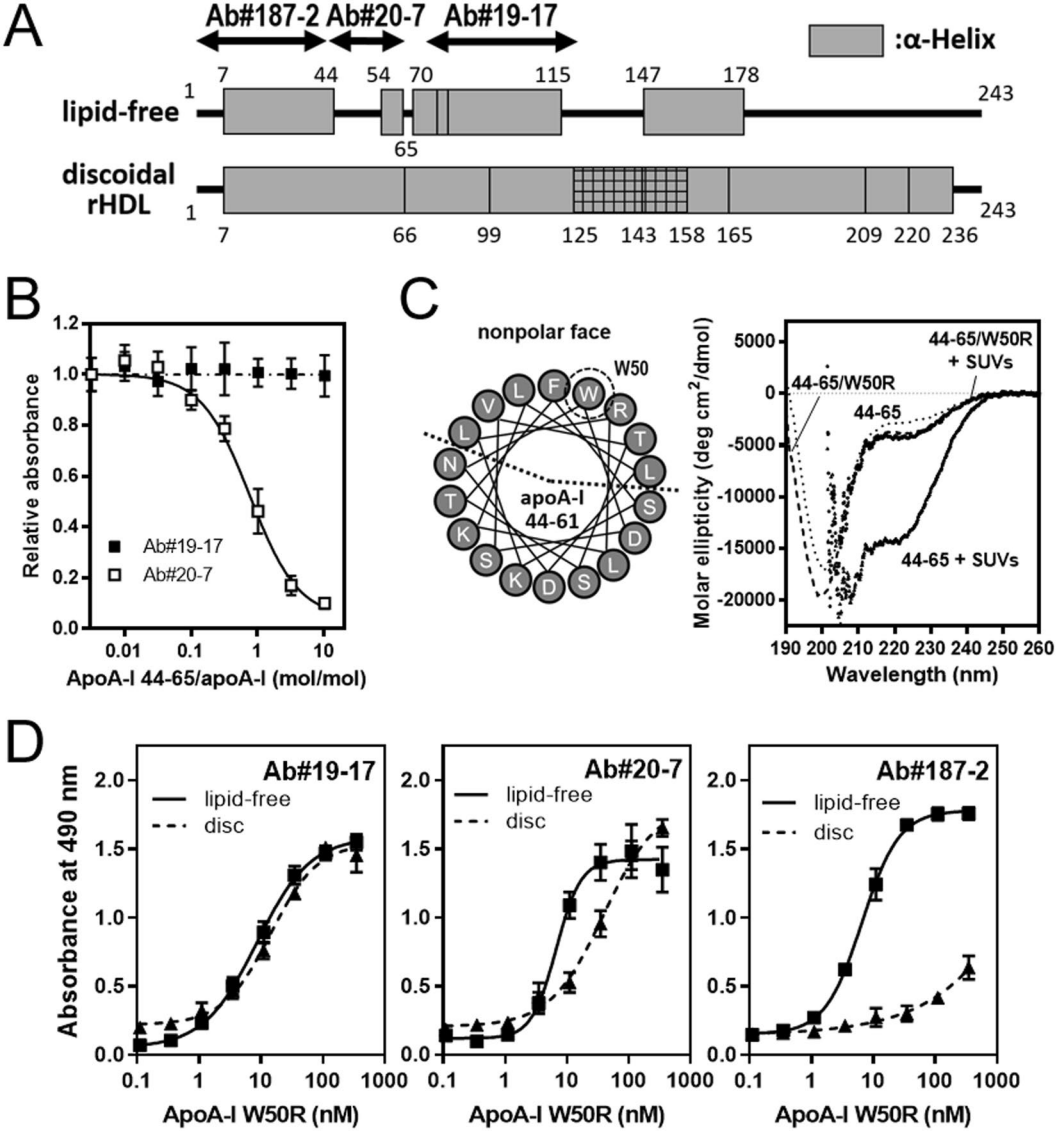
Effect of W50R mutation in apoA-I on reactivity of mAbs. (**A**) Locations of α-helical segments in lipid-free and discoidal HDL apoA-I^21, 54^. The cylinders represent α-helices, and the lines indicate random coil structure. The crosshatched region (residues 125–158) indicates coexisting helical and disordered loop conformations. The epitope-containing segments for Ab#19-17, Ab#20-7, and Ab#187-2 were shown as arrowed lines. (**B**) Competitive ELISA for binding of Abs#19-17 and Ab#20-7 to lipid-free apoA-I (35 nM) with increasing concentrations of apoA-I 44–65 peptide. The absorbance at 490 nm in the absence of apoA-I 44–65 was normalized to 1.0. (**C**) Helical wheel diagrams of residues 44–65 of apoA-I. Trp-50 is located on the center of non-polar face of amphipathic α-helix (left). Far-UV CD spectra of apoA-I 44–65 and W50R variant peptides in the absence or presence of egg phosphatidylcholine small unilamellar vesicles (SUVs) (right). (**D**) Sandwich ELISA for binding of mAbs to full-length apoA-I W50R variant in the lipid-free state or on discoidal HDL.

Figure 5A shows the locations of α-helical segments in lipid-free and discoidal HDL apoA-I determined by hydrogen-deuterium exchange analyses^21, 54^. The transition of apoA-I from the lipid-free to the HDL-bound state is accompanied by a conformational change from random coil to α-helix in the N-terminal (residues 45–53 and 66–69), central (residues 116–146), and C-terminal (residues 179–236) regions. In contrast, crystal structure of C-terminal truncated apoA-I demonstrated that the N-terminal 43 residues form a major helix (residues 7–34) and a minor helix (residues 37–41)^55^, indicating the possibility that even within residues 7–44 shown as forming α-helix in Fig. 5A, there are certain residues to undergo structural transition upon HDL formation. Thus, we hypothesized that Ab#20-7 and Ab#187-2 cannot recognize epitope region (within residues 44–65 and 1–43, respectively) when forming α-helical structure in lipidated apoA-I.

To verify this hypothesis, we focused on the structural transition of residues 44–65 because this region contains an obvious random coil segment in lipid-free apoA-I revealed by both hydrogen-deuterium exchange analysis (residues 45–53)^54^ and x-ray crystallography (residues 44–54)^55^. We first used apoA-I 44–65 synthetic peptide in a competitive ELISA because this peptide is predominantly in a random coil structure in solution^56^. As shown in Fig. 5B, binding of Ab#20-7 to lipid-free apoA-I was stoichiometrically competed by apoA-I 44–65 peptide (the molar ratio of the 44–65 peptide/apoA-I at IC_50_ was close to 1.0 mol/mol) whereas no competition was observed for Ab#19-17. This clearly indicates that Ab#20-7 recognizes residues 44–65 of apoA-I in a random coil structure.

We next introduced the mutation W50R into apoA-I to disrupt α-helical structure within residues 44–65 when the protein is in a lipid-bound state. The W50R mutation, known as an amyloidogenic mutation in apoA-I^57, 58^, places the strongly basic arginine residue into the nonpolar face of the amphipathic α-helix in residues 44–65 (Fig. 5C left), leading to destabilization of the helical structure. Indeed, far-UV CD measurements demonstrated that while apoA-I 44–65 peptide transforms from random coil to α-helical structure upon lipid binding, the W50R mutation strongly prevents α-helix formation of the 44–65 peptide (Fig. 5C right).

Figure 5D shows sandwich ELISA for binding of Ab#19-17, Ab#20-7, and Ab#187-2 to the apoA-I W50R variant in lipid-free or discoidal HDL forms. It should be noted that although the W50R mutation disrupts local helical structure in residues 44–65, it does not significantly affect entire helical structure and protein-lipid interactions in full-length apoA-I^59^. Indeed, size distributions of discoidal HDL particles formed with full-length apoA-I were almost identical in wild-type and the W50R variant (the hydrodynamic diameters were 9.2 ± 2.1 and 9.4 ± 2.1 nm for wild-type and the W50R apoA-I, respectively). All three mAbs recognize lipid-free apoA-I W50R similarly with *K*_D_ values of 7–10 nM, indicating that the W50R mutation does not affect reactivity of three mAbs against lipid-free apoA-I. Importantly, the W50R mutation restored the reactivity of Ab#20-7 against apoA-I in discoidal HDL-bound state, whereas almost no effect on the reactivity was observed for Ab#19-17 neither Ab#187-2 compared to wild-type apoA-I discoidal HDL (Fig. 4B). These results support the hypothesis that Ab#20-7 recognizes residues 44–65 of apoA-I only in a random coil structure.

To further confirm the secondary structure specificity for apoA-I recognition of mAbs, we selected another mAb, Ab#126-7 whose epitope is within residues 44–65 in apoA-I (Fig. S5A). Similarly to the case of Ab#20-7, this mAb recognized lipid-free apoA-I and plasma HDL well (*K*_D_ values are 2.7 and 3.6 nM, respectively), but did not recognize reconstituted discoidal HDL particles (Fig. S5B). In the W50R variant, in contrast, Ab#126-7 was able to recognize discoidal HDL with comparable affinity (*K*_D_ of 11 nM) to the case of plasma HDL (Fig. S5C). Based on these results, it is plausible that anti-apoA-I mAbs having epitopes within residues 44–65 can detect the structural transition from random coil to α-helix in apoA-I.

## Conclusion

In this report, we have developed novel anti-apoA-I mAbs targeting epitopes within the N-terminal residues in apoA-I that undergo structural transition during the formation and maturation of HDL particles. Using protein engineering with site-directed mutagenesis, we demonstrated that these antibodies specifically recognize the epitope region of apoA-I in a random coil state, but not in α-helical structure. In particular, since residues 45–53 are thought to transform from random coil to α-helical structure upon HDL formation, the mAbs targeting residues 44–65 found in this study may have potential as a structure-specific antibody to assess structure and function of HDL subpopulations.

## Materials and Methods

### Buffers

The buffers used in this study, abbreviated as PB, PBS, G-PBS, T-PBS, M-PBS, and PVG-PBS, are reviewed in detail in Supplementary Information.

### Preparation of recombinant apoA-I proteins and peptide

To introduce an additional N-terminal Cys residue or Trp50Arg mutation in human apoA-I, *apoA-I* genes were engineered by PCR overlap extension method with primers, 5′-GGTACCACGCGGATCCTGTGATGAACCACCACAG-3′ and 5′-CTGTGGTGGTTCATCACAGGATCCGCGTGGTACC-3′ for Cys-apoA-I and 5′-AAGCTCCTTGACAACCGTGACAGCGTGACCTCC-3′ and 5′-GGAGGTCACGCTGTCACGGTTGTCAAGGAGCTT-3′ for apoA-I Trp50Arg, respectively. The mutant genes obtained, as well as *WT apoA-I* gene, were subcloned in pET32a+ vector (Novagen, Madison, WI) and expressed in *E*. *coli* BL21 Star (DE3) to produce the target proteins fused with thioredoxin. Cleavage of these fusions with thrombin and subsequent purification^18^ provided the desired apoA-I variants with two extra amino acids, Gly-Ser, at the N-terminus. The purity of apoA-I preparations was assessed by SDS-PAGE and reverse-phase HPLC (Fig. S6). Protein molecular mass was determined by MALDI-TOF MS analysis (Fig. S7). The apoA-I 44–65 peptide was synthesized by the solid-phase method with Fmoc chemistry^58, 60^. These apoA-I variants and peptide were dialyzed from 6 M guanidine hydrochloride solution into PBS before use.

### Generation of monoclonal antibody

All experiments on animals were carried out in accordance with guidelines and regulations established in Kobe Pharmaceutical University, and all experimental protocols were approved by the Institutional Animal Care and Use Committee. ***Immunization***. Two groups (A and B) of five female BALB/c mice (8 weeks of age; Japan SLC, Hamamatsu, Japan) were immunized with the recombinant apoA-I (for A) or KLH-conjugated apoA-I (see Supplementary Information) (for B) at two-week intervals. Immunogens (25 μg/mouse) were subcutaneously injected with Freund’s complete adjuvant (primary immunization) or incomplete adjuvant (booster immunizations), emulsified with sterile saline (1:1; 0.2 ml/mouse)^61, 62^. Seven days after the third booster, blood samples were collected from each individual, and the titer of serum antibodies was determined by ELISA (see Supplementary Information). For group A, two mice with higher titer received intraperitoneal and intrasplenic injections of apoA-I (totally 50 μg) in saline^61, 62^, and after three days, splenocytes therefrom were prepared for cell fusions. For group B, the mice were boosted once more with intact apoA-I without KLH conjugation after six months, and serum titer were checked again. Then, apoA-I (50 μg) was injected similarly into two mice with higher titer, and splenocytes were prepared. ***Cell fusion and antibody production***. Cell fusion^46^ was performed as described previously^61, 62^. In brief, splenocytes from the immunized mice (1–2 × 10^8^ cells) and 1/5 number of P3/NS1/1-Ag4-1 (NS-1) myeloma cells^63^ were fused with a 40% polyethylene glycol 4000 solution (1 ml)^61, 62^. The fused cells were cultured in a HAT medium supplemented with 5% hybrido hybridoma growth factor (Recenttec, Taipei City, Taiwan) under 5% CO_2_/95% air at 37 °C for *ca*. two weeks. The antibody-secreting hybridomas were screened by ELISA (see Supplementary Information), expanded in HT medium, and then cloned by limiting dilution. The cloned hybridomas were grown in a large scale and culture supernatants were used for initial characterization of the mAbs. Selected mAbs (Ab#19-17, Ab#20-7, Ab#124-2, Ab#126-7, and Ab#187-2) were produced as ascites fluids by intraperitoneal injection of relevant hybridomas into male BALB/c mice pretreated with pristane (0.5 ml/mouse). Mabs therein were partially purified as the IgG fractions by a HiTrap Protein G HP column (GE Healthcare Japan, Tokyo, Japan).

### Dot blotting

ApoA-I variants blotted on a nitrocellulose transfer membrane were probed with anti-apoA-I mAbs followed by POD-conjugated AffiniPure goat anti-mouse IgG antibody (Jackson ImmunoResearch, Inc., West Grove, PA) and ECL Prime Western Blotting Detection Reagent (GE Healthcare Japan). Signals were visualized by a C-DiGit Blot Scanner (M&S TechnoSystems, Osaka, Japan) using apoA-I and PBS as control.

### Preparation of HDL particles

The total HDL fraction (1.063 < d < 1.21 g/ml) was purified by sequential ultracentrifugation from normolipidemic human plasma as described previously^64^. Reconstituted discoidal HDL was prepared from POPC and apoA-I by cholate dialysis as reported earlier^65^. Spherical HDL was prepared by co-sonication of POPC, triolein, and apoA-I and isolated by sequential ultracentrifugation as described^11^. Dynamic light scattering measurements on a Zetasizer Nano ZS (Malvern, Malvern, UK) demonstrated the homogeneous distribution of discoidal and spherical HDL particles with an average diameter of 10 nm (Fig. S4B).

### Biotinylation of apoA-I, apoA-I fragmemt, and mAbs

For biotinylation of Cys-apoA-I, 10-fold molar excess of tris(2-carboxyethyl)phosphine HCl (TCEP, Thermo Fisher Scientific) was added to a solution of Cys-apoA-I in PBS, and the mixture was incubated for 1.5 h at room temperature to generate sulfhydryl groups. Then, 10-fold molar excess of biotin-PEAC_5_-maleimide (Dojindo, Kumamoto, Japan), dissolved in dimethyl sulfoxide (DMSO), was added to the reduced Cys-apoA-I in PBS (1 ml), and the mixture was incubated overnight at 4 °C. For biotinylation of plasma apoA-I, recombinant wild-type and 1–83 fragment of apoA-I, and mAbs, three- (for mAbs) or 10-fold (for apoA-I proteins) molar excess of EZ-Link NHS-LC-Biotin (Thermo Fisher Scientific), dissolved in DMSO, was added to the target protein in PBS (500 μl), and the mixture was incubated for 2 h at 4 °C. After these reactions, unreacted reagents were removed by extensive dialysis at 4 °C in PBS. The degree of labeling was determined by SensoLyte HABA biotin quantitation kit (AnaSpec, Fremont, CA).

### Single-antibody ELISA

Microwells of the Costar microplates were coated overnight at 4 °C with 25 μg/ml streptavidin (Funakoshi, Tokyo, Japan) in PBS (100 μl/well), and then blocked by incubating with 1% Block Ace (DS Pharma Biomedical, Osaka, Japan) for 60 min at 37 °C. The wells were washed with T-PBS and then incubated with biotinylated apoA-I samples diluted with G-PBS (100 μl/well) for 30 min at 37 °C. After washing with T-PBS, serially diluted mAbs in G-PBS (0–66.7 nM) were added (100 μl/well) and incubated for 30 min at 37 °C. The plates were washed again with PBS, and then a POD-conjugated AffiniPure goat anti-mouse IgG antibody (Jackson ImmunoResearch), diluted at 1:5000 in G-PBS, was added (100 μl/well) and incubated for 30 min at 37 °C. After washing the wells with T-PBS, the bound POD activity was determined colorimetrically by the absorbance at 490 nm with absorbance at 620 nm for reference using Infinite 200 PRO microplate reader (Tecan Japan, Kawasaki, Japan). ELISA data were fitted by nonlinear regression to a sigmoidal dose-response curve with variable slope model using the GraphPad Prism program.

### Sandwich ELISA

Microwells of the Costar microplates were coated by incubating overnight at 4 °C with 100 μl/well of the purified Ab#124-2 in PBS (100 μg/mL), and then blocked with Block Ace. The wells were washed with T-PBS and then incubated for 30 min at 37 °C with apoA-I samples (lipid-free, reconstituted discoidal and spherical HDL particles, and plasma HDL) at 0.11–350 nM in 100 μl/well, which were serially diluted with G-PBS. After washing with PBS (without detergent), biotinylated Ab#19-17, Ab#20-7, or Ab#187-2 in G-PBS (100 μl/well) was added and incubated for 30 min at 37 °C. Wells were washed with T-PBS, and then incubated for 30 min at 37 °C with 1.0 μg/ml POD-conjugated streptavidin (Jackson ImmunoResearch) in G-PBS (100 μl/well). After washing, the bound POD activity was measured as described above.

### Competitive ELISA

Microwells of the Costar microplates were coated similarly with 10 μg/ml purified Ab#19-17 or Ab#20-7, and then blocked with 1% Block Ace. Solutions of a constant amount of biotinylated wild-type apoA-I (35 nM) and varying amounts of apoA-I 44–65 peptide (0.11–350 nM) diluted with G-PBS, were mixed with 1:1 by volume, and then 100 μl aliquots of which were added to the wells and incubated for 30 min at 37 °C. After washing with T-PBS, the POD-conjugated streptavidin was reacted and bound enzyme activity was determined as described above.

### BLI analysis

BLI experiments were performed using OctetRED 96 system (forteBIO, Pall Life Sciences, Menio Park, CA). Microwells in 96 well black polystyrene plates were used as the reaction chamber, and all association and dissociation reactions were performed in 200 μl solution. Streptavidin-coated sensor chips were dipped in PBS for 600 s for hydration, and the biotinylated mAbs (10 μg/ml) was immobilized on the sensor chips for 300 s. BLI sensorgrams were measured in three steps: baseline (60 s), association to the sensor chips (180 s), and dissociation from the sensor chips (180 s). ApoA-I in the lipid-free form, on reconstituted discoidal HDL, and plasma HDL were set to 44–350 nM in PBS. Signals were monitored and recorded every 0.2 s with sample plates being continuously shaken at 1,000 rpm to eliminate mass transport effect. Sensorgram raw data were processed by Octet software, and evaluated by BIAevaluation Software Version 4.1 (GE healthcare Japan). The Langmuir model for 1:1 binding were used to solve simultaneously for association (*k*_on_) and dissociation (*k*_off_) rates.

### CD spectroscopy

Far-UV CD spectra were recorded from 190 to 260 nm at 25 °C using a Jasco J-1500 spectropolarimeter (JASCO, Tokyo, Japan). The apoA-I solutions of 50 μg/ml in Tris-HCl buffer (pH 7.4) were subjected to CD measurements in a 2 mm quartz cuvette. The α-helical content was derived from the molar ellipticity at 222 nm ([*θ*]_222_) using the equation: % α-helix = [(−[*θ*]_222_ + 3000)/(36000 + 3000)] × 100, assuming that all the protein in plasma HDL is apoA-I.

### Statistical analysis

Data were analyzed via the unpaired Student’s *t* test or one-way analysis of variance with Dunnett’s test. Results were regarded as significant for *P* < 0.05. All data are means ± SEM unless otherwise noted.

## Electronic supplementary material

Supplementary information
