# Supplementary material for: Immunochemical Approach for Monitoring of Structural Transition of ApoA-I upon HDL Formation Using Novel Monoclonal Antibodies
Source: Sci Rep. 2017 Jun 7;7:2988. doi: 10.1038/s41598-017-03208-8 (PMC5462821; doi:10.1038/s41598-017-03208-8)
Supplement: Supplementary file 1 — Supplementary information [file 41598_2017_3208_MOESM1_ESM.pdf]

Supplementary Information

Immunochemical Approach for Monitoring of Structural Transition of ApoA-I upon HDL Formation Using Novel Monoclonal Antibodies

Hitoshi Kimura, Shiho Mikawa, Chiharu Mizuguchi, Yuki Horie, Izumi Morita, Hiroyuki Oyama, Takashi Ohgita, Kazuchika Nishitsuji, Atsuko Takeuchi, Sissel Lund-Katz, Kenichi Akaji, Norihiro Kobayashi, and Hiroyuki Saito

Results and Discussion

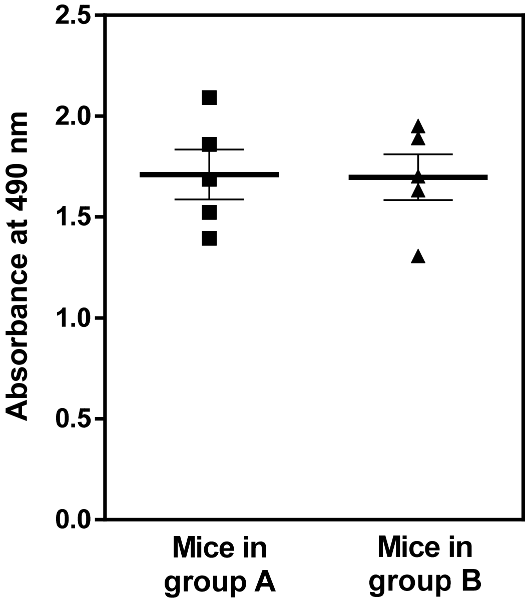

Figure S1. Serum titer for anti-apoA-I antibodies in the mice after immunization. Blood (10–50 μl) was collected from each mice individual (#1-5) after the third booster immunization with apoA-I (group A) or apoA-I-KLH conjugate (group B). Sera were diluted (1:5000) with G-PBS and submitted to the ELISA described below. Enzyme reaction for the color development was performed for 15 min at room temperature.

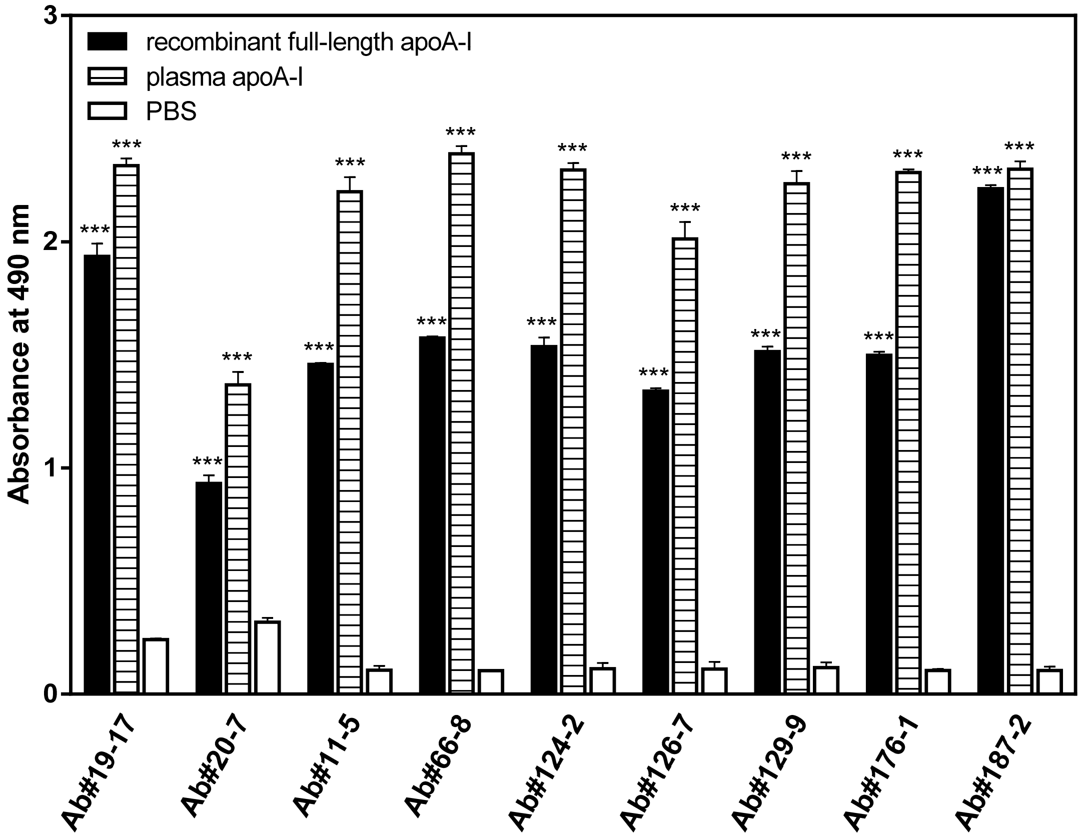

Figure S2. Reactivity of anti-apoA mAbs to recombinant full-length apoA-I and plasma apoA-I. Biotinylated recombinant full-length apoA-I or plasma apoA-I purified from human plasma HDL[1] (0.3 μM), or PBS was added to 96-well microplates coated with streptavidin. After washing, 500-fold diluted hybridoma supernatants were added to the wells and incubated. After washing, POD-conjugated anti-mouse IgG antibody diluted at 1:5000 was added and incubated. After washing, bound POD activity was determined colorimetrically. ***$P < 0.001$ versus PBS.

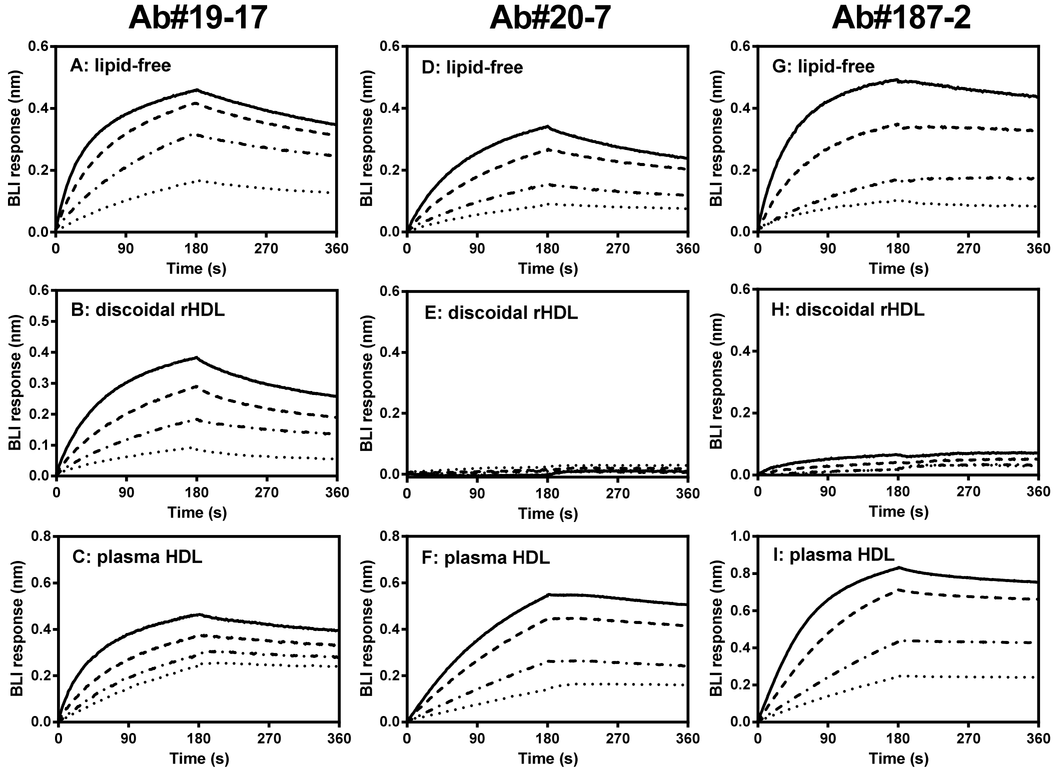

Figure S3. Bio-layer interferometry sensorgrams for binding of apoA-I to immobilized antibody on a sensor chip. Processed sensorgrams for association and dissociation of apoA-I in the lipid-free state (A, D, and G), on reconstituted discoidal HDL (B, E, and H), and human plasma HDL (C, F, and I). Biotinylated Ab#19-17, Ab#20-7, and Ab#187-2 were loaded to streptavidin biosensor chips. MAb-immobilized chips were dipped into apoA-I solutions at a range of concentrations for association, and then into PBS for dissociation.

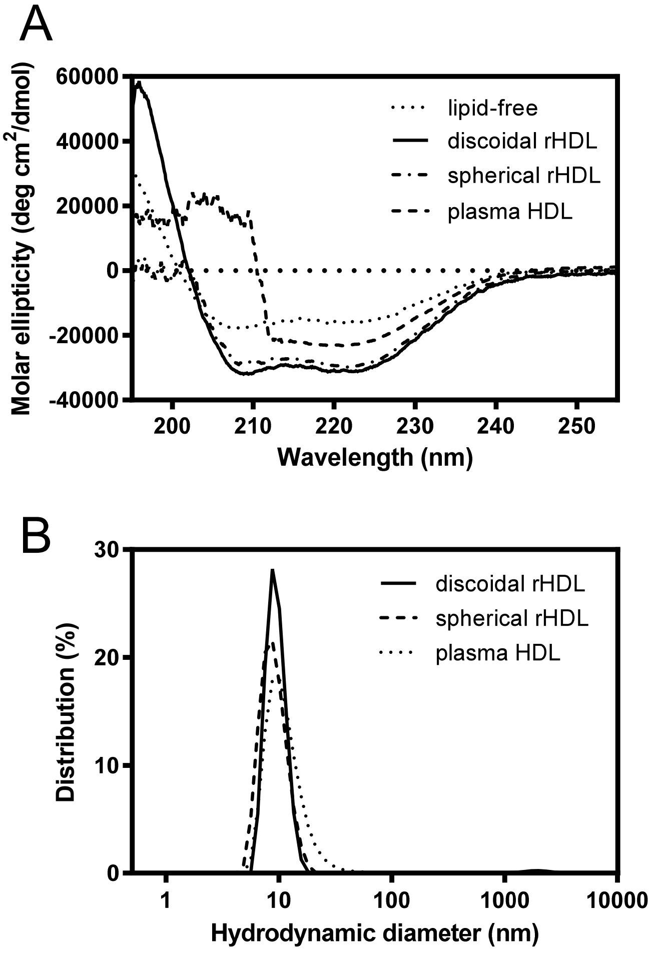

Figure S4. Characterization of reconstituted discoidal or spherical HDL, and human plasma HDL. (A) Far-UV CD spectra of apoA-I in the lipid-free state, on reconstituted discoidal HDL and spherical HDL, and human plasma HDL. The α-helix contents determined from signals at 222 nm are 48, 88, 84, and 67% for apoA-I in the lipid-free state, on discoidal rHDL, spherical rHDL, and human plasma HDL, respectively, assuming that all the protein in plasma HDL is apoA-I. Global secondary structure analysis of lipid-free apoA-I using the spectra from 210 to 260 nm in the CDNN neural network yields approximately 51% α-helix, 11% β-strand, and 38% random coil, consistent with the previous report[2]. It should be noted that when applied to lipid-bound proteins, the resulting calculations can produce considerable artefacts due to light scattering and absorption flattening[3]. (B) Particle size distribution of discoidal rHDL, spherical rHDL, and plasma HDL determined by dynamic light scattering measurements on a Zetasizer Nano ZS (Malvern). The data were represented as volume-based distributions.

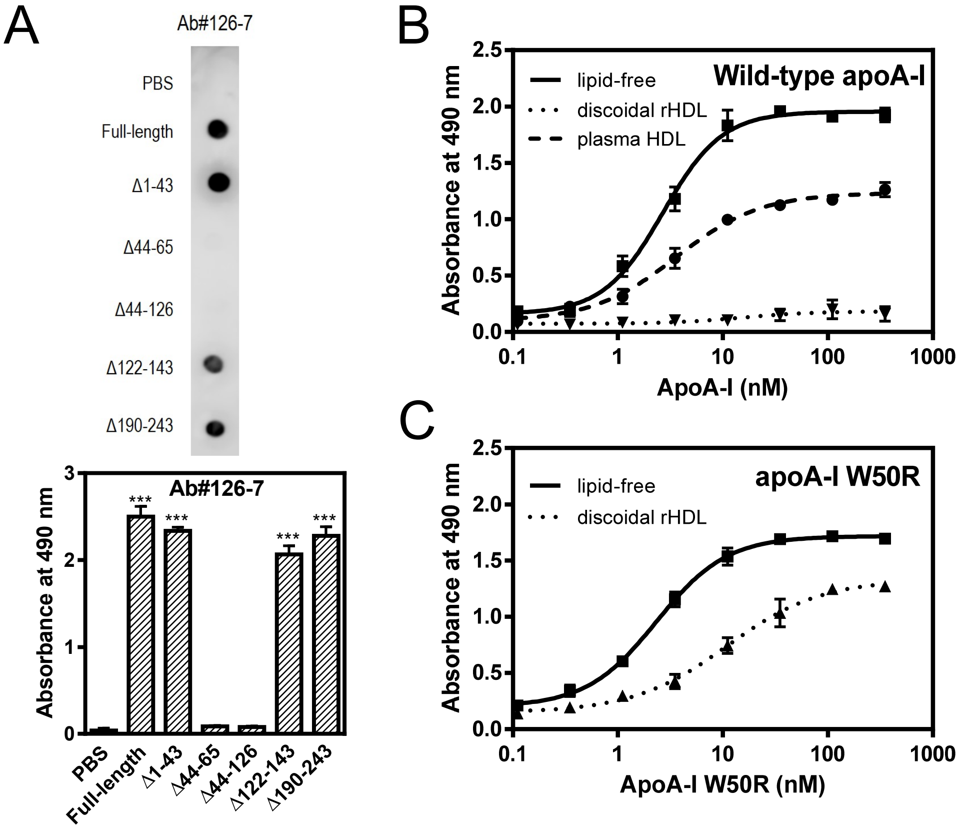

Figure S5. Effect of W50R mutation on reactivity of Ab#126-7 against lipid-free and HDL-bound apoA-I. (A) Dot blotting (top) and reactivity in ELISA (bottom) with apoA-I deletion mutants for Ab#126-7 to determine epitope. (B) Sandwich ELISA for binding of Ab#126-7 to apoA-I in the lipid-free state or on discoidal rHDL, and human plasma HDL. Similarly to the Ab#20-7, Ab#126-7 showed very low signals against disc-bound apoA-I. (C) Sandwich ELISA for binding of Ab#126-7 to apoA-I W50R variant in the lipid-free state or on discoidal rHDL. The reactivity of Ab#126-7 against disc-bound apoA-I was recovered by the W50R mutation. ***$P <$ 0.001 versus PBS.

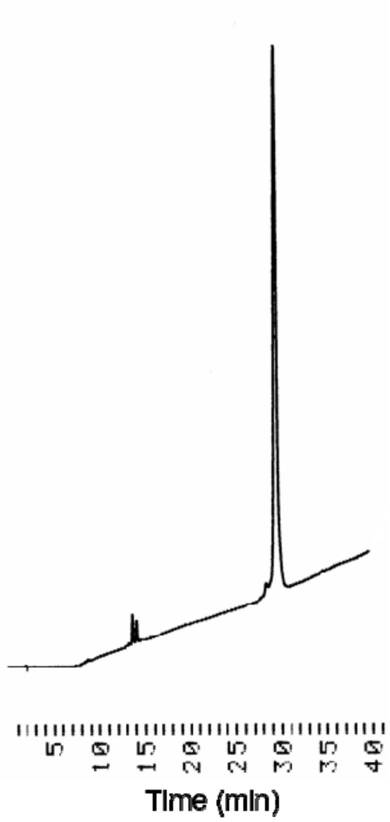

Figure S6. Reverse-phase HPLC profiles of recombinant full-length apoA-I. The sample was eluted with a linear gradient of acetonitrile and water containing 0.05% trifluoroacetic acid on a TOSHO TSKgel Octadecyl-4PW column.

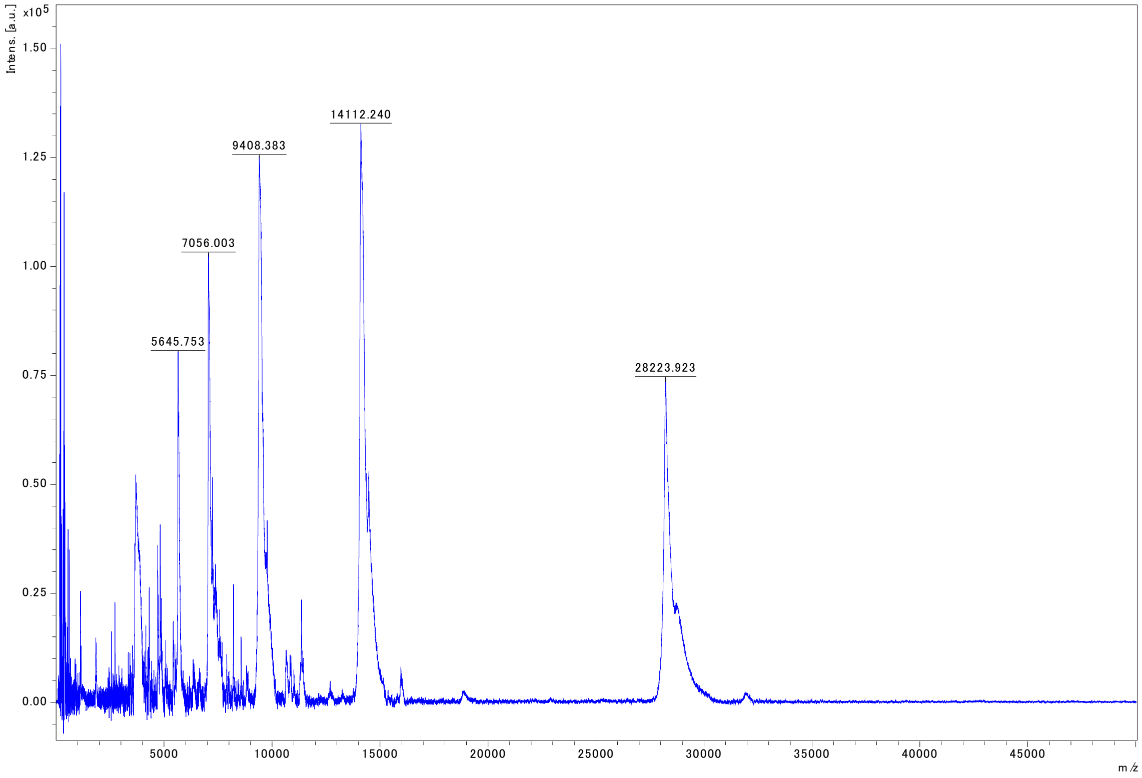

Figure S7. MALDI-TOF MS spectrum of recombinant full-length apoA-I. MALDI-TOF MS analysis was performed using a microflex instrument (Bruker Daltonics, Bremen, Germany) in the positive linear ion mode. Spectrum was calibrated externally using a standard protein mixture (Protein calibration standard I and II, Bruker). The ion observed at m/z 28223.9 was estimated to be the protonated molecule $(M + H)^+$: molecular mass of the recombinant full-length apoA-I is calculated to be 28222.75.

Table S1. Kinetic parameters for binding of apoA-I to Ab#19-17, Ab#20-7, and Ab#187-2 determined by BLI measurements.

#19-17

apoA-I	k_{on}	S.E. (k_{on})	k_{off}	S.E. (k_{off})	K_D	S.E. (K_D)
	$10^4\ M^{-1}\ s^{-1}$		$10^{-4}\ s^{-1}$		*nM*	
lipid-free	6.5	0.021	14.0	0.060	21.6	0.059
discoidal rHDL	3.9	0.013	20.7	0.050	53.6	0.29
plasma HDL	5.7	0.022	5.4	0.052	9.4	0.11

#20-7

apoA-I	k_{on}	S.E. (k_{on})	k_{off}	S.E. (k_{off})	K_D	S.E. (K_D)
	$10^4\ M^{-1}\ s^{-1}$		$10^{-4}\ s^{-1}$		*nM*	
lipid-free	3.2	0.015	16.1	0.047	50.0	0.37
discoidal rHDL	N.D.	N.D	N.D.	N.D.	N.D.	N.D.
plasma HDL	2.1	0.010	4.1	0.054	19.7	0.35

#187-2

apoA-I	k_{on}	S.E. (k_{on})	k_{off}	S.E. (k_{off})	K_D	S.E. (K_D)
	$10^4\ M^{-1}\ s^{-1}$		$10^{-4}\ s^{-1}$		*nM*	
lipid-free	7.6	0.024	3.6	0.053	4.8	0.085
discoidal rHDL	N.D.	N.D	N.D.	N.D.	N.D.	N.D.
plasma HDL	4.7	0.013	4.3	0.031	9.1	0.080

Materials and Methods

Buffers. The following abbreviations were used for the buffers employed in this study: PB, 50 mM sodium phosphate buffer (pH 7.3); PBS, PB containing 9.0 g/l NaCl; G-PBS, PBS containing 1.0 g/l gelatin; T-PBS, PBS containing 0.050% (v/v) Tween 20, M-PBS: PBS containing 20 g/l skim milk; and PVG-PBS, G-PBS containing 1.0 g/l polyvinyl alcohol (average polymerization degree = 500).

Preparation of apoA-I-KLH conjugate. 1-Ethyl-3-(3-dimethylaminopropyl) carbodiimide HCl (EDC) (25 mg) was added to a mixture of the recombinant apoA-I (0.85 mg) and keyhole limpet hemocyanin (KLH; EMD Millipore, San Diego, CA) (1.5 mg) dissolved in PB (1.5 ml). The resulting solution was stirred at room temperature for 2 h, then at 4 °C overnight. After dialysis against cold PB for 2 days, the desired KLH-conjugated apoA-I was obtained as a PB solution, which was stored at –20 °C until use.

Determination of the titer for serum anti-apoA-I antibodies.

• **Biotinylation of apoA-I and its N-terminal 1-83 fragment.** A solution of Biotin(Long Arm)NHS (ThermoFisher Scientific, Waltham, MA) (10 mol equiv) in 1,4-dioxane : *N*, *N*-dimethylformamide (1 : 1, 0.50 ml) was added to a solution of the recombinant apoA-I (4.0 mg) in PB (1.0 ml). This mixture was stirred at room temperature for 60 min, then at 4 °C overnight. Dialysis against cold PB for 2 days provided biotinylated apoA-I as PB solutions.

• **ELISA.** Microwells of Costar 96-well EIA/RIA microplates (No. 3590; Coning, Corning, NY) were coated with an affinity-purified goat anti-mouse IgG antibody ("second antibody": Jackson ImmunoResearch, West Grove, MA), and then blocked with M-PBS[4,5]. The wells were washed 3 times with T-PBS, and sera from immunized mice, diluted with G-PBS, were added to the wells (100 µl/well). After incubating at 37 °C for 60 min, the solutions were removed and the wells were washed similarly. A solution of biotinylated full-length apoA-I in PVG-PBS (300 ng/ml) or 1-83 fragment in GPB (100 ng/ml) was then added (100 µl/well) and incubated at 37 °C for 60 min. After washing, a solution of POD-labeled streptavidin (Jackson ImmunoResearch) in PVG-PBS (200 ng/ml) was added (100 µl/well) and incubated at 37 °C for 30 min. The wells were washed and the bound POD activity was determined colorimetrically using *o*-phenylenediamine as a hydrogen donor[2,3]. The absorbance at 490 nm was measured using a microplate reader.

Screening of hybridomas secreting anti-apoA-I antibodies

• **Screening of hybridomas derived from the mice in group A.** The recombinant apoA-I (0.85 mg) was conjugated with bovine serum albumin (BSA; 1.5 mg) using EDC (25 mg) as described above. Microwells of the Costar microplates (No. 3590) were incubated with a solution of this conjugate in 0.10 M carbonate buffer (pH 8.7) (10 μg/ml; 100 μl/well) overnight at room temperature. After washing, the wells were blocked with M-PBS[4,5]. Hybridoma supernatants, 6-fold diluted with G-PBS, were added to the wells (120 μl/well) and incubated at 37 °C for 60 min. The wells were washed and incubated with a solution of goat POD-labeled anti-mouse IgG antibody (Jackson ImmunoResearch) in G-PBS (160 ng/ml; 100 μl/well) at 37 °C for 30 min. After washing, the bound POD activity was determined as described above.

• **Screening of hybridomas derived from the mice in group B.** Microwells of the Costar microplates (No. 3590) were coated with the second antibody and blocked as described above. After washing, hybridoma supernatants, 6-fold diluted with G-PBS, were added to the wells (120 μl/well) and incubated at 37 °C for 60 min. The wells were washed and incubated with a solution of POD-labeled protein L (Jackson ImmunoResearch) in PVG-PBS (1.0 μg/ml; 100 μl/well) at 37 °C for 30 min. After washing, the bound POD activity was determined as described above.

References

1. Saito, H. *et al.* Domain structure and lipid interaction in human apolipoproteins A-I and E, a general model. *J. Biol. Chem.* **278**, 23227-23232 (2003).
2. Brubaker, G., Peng, D. Q., Somerlot, B., Abdollahian, D. J. & Smith, J. D. Apolipoprotein A-I lysine modification: effects on helical content, lipid binding and cholesterol acceptor activity. *Biochim. Biophys. Acta* **1761**, 64-72 (2006).
3. Kelly, S. M., Jess, T. J. & Price, N. C. How to study proteins by circular dichroism. *Biochim. Biophys. Acta* **1751**, 119-139 (2005).
4. Kobayashi, N. *et al.* A monoclonal antibody-based enzyme-linked immunosorbent assay for human urinary cotinine to monitor tobacco smoke exposure. *Anal. Methods* **3,** 1995-2002 (2011).
5. Oyama, H. *et al.* Gaussia luciferase as a genetic fusion partner with antibody fragments for sensitive immunoassay monitoring of clinical biomarkers. *Anal. Chem.* **87,** 12387-12395 (2015).
